# Accounting for Heterogeneity: Mixed-Effects Models in Resting-State EEG Data in a Sample of Tinnitus Sufferers

**DOI:** 10.1007/s10548-020-00772-7

**Published:** 2020-04-23

**Authors:** Constanze Riha, Dominik Güntensperger, Tobias Kleinjung, Martin Meyer

**Affiliations:** 1 Chair of Neuropsychology, Department of Psychology, University of Zurich, Binzmühlestr. 14/25, 8050 Zurich, Switzerland; 2 Research Priority Program “ESIT - European School of Interdisciplinary Tinnitus Research”, Zurich, Switzerland; 3 Department of Otorhinolaryngology, University Hospital Zurich, Zurich, Switzerland

**Keywords:** Electroencephalography, Resting-state, Mixed-effect model, Tinnitus, Oscillation

## Abstract

In neuroscience, neural oscillations and other features of brain activity recorded by electroencephalography (EEG) are typically statistically assessed on the basis of the study’s population mean to identify possible blueprints for healthy subjects, or subjects with diagnosable neurological or psychiatric disorders. Despite some inter-individual similarities, there is reason to believe that a discernible portion of the individual brain activity is subject-specific. In order to encompass the potential individual source of variance in EEG data and psychometric parameters, we introduce an innovative application of linear mixed-effects models (LMM) as an alternative procedure for the analysis of resting-state EEG data. Using LMM, individual differences can be modelled through the assumptions of idiosyncrasy of all responses and dependency among data points (e.g., from the same subject within and across units of time) via random effects parameters. This report provides an example of how LMM can be used for the statistical analysis of resting-state EEG data in a heterogeneous group of subjects; namely, people who suffer from tinnitus (ringing in the ear/s). Results from 49 participants (38 male, mean age of 46.69 ± 12.65 years) revealed that EEG signals were not only associated with specific recording sites, but exhibited regional specific oscillations in conjunction to symptom severity. Tinnitus distress targeted the frequency bands beta3 (23.5–35 Hz) and gamma (35.5–45 Hz) in right frontal regions, whereas delta (0.5–4 Hz) exhibited significant changes in temporal-parietal sources. Further, 57.8% of the total variance in EEG power was subject-specific and acknowledged by the LMM framework and its prediction. Thus, a deeper understanding of both the underlying statistical and physiological patterns of EEG data was gained.

## Introduction

Resting-state electroencephalography (EEG) has a long tradition in the field known in the present-day as neuroscience (Berger 1929). EEG studies aim to examine the relationships between behavioral measures and their underlying neural mechanisms. Dependent on the question, these relationships are explored in various testing conditions and subject populations. Typically, after pre-processing the recordings, the generated EEG data of several subjects from a specific population are collapsed to make them amenable for analysis and to draw inference about specific features in the EEG signal (e.g., general oscillatory patterns). Previously, researchers have used such features in attempts to identify general blueprints of brain activity, the shared patterns or contrasting abnormalities of which within a group of subjects would allow the assignment of individuals to a certain population (e.g., to healthy subjects or to subjects with diagnosable neurological or psychiatric disorders). Yet, despite gross inter-individual similarities, there is reason to believe that a discernible portion of the individual spontaneous cortical activity is subject-specific (Valizadeh et al. 2019); that is, it varies substantially from one subject to another (Barch et al. 2013; Finn et al. 2015; Valizadeh et al. 2019) and can be recognized and attributed to that same individual several months later (Chu et al. 2012; Cox et al. 2018).

The preferred methodological approaches to analyze EEG derived data from a sample of individuals or to contrast two populations are usually ordinary linear regression or multivariate analysis of variance (ANOVA). Both of these standard statistical models share one assumption: All data points are independent and identically distributed (iid).^1^ However, many studies violate this assumption by collecting measures from the same subjects within (i.e., single acquisition) and across units of time (e.g., longitudinal studies). Consequently, the reported significance tests based on these standard statistical models suffer from an increased Type 1 error rate (i.e., the probability of reporting false positive results is inflated) (Barr et al. 2013; Judd et al. 2012; Matuschek et al. 2017). With reference to EEG studies, already a single recording of a subject’s brain activity corresponds to an idiosyncratic factor that affects all oscillatory responses of each electrode from the same subject and thus renders the recorded data interdependent rather than independent. This discrepancy can be solved statistically using linear mixed-effects models (LMM): “Mixed-effects models are primarily used to describe relationships between a response variable and some covariates in data that are grouped according to one or more classification factors” (Pinheiro and Bates 2000, p. 3). Ecology (Houslay and Wilson 2017; Zuur 2009), (psycho-) linguistics (Baayen et al. 2008), biology (Houslay and Wilson 2017; Zhang et al. 2010), and the neurosciences (Cornew et al. 2012) are only some of the disciplines in which LMMs have proven useful in the analysis of heterogeneous samples while capturing dependencies between data points via random-effects.

### Departure from the Standard: Linear Mixed-Effects Models for EEG Studies

Conceptually, *random-effects* resolve the non-independence by assuming that the parameters follow a random distribution (usually a normal distribution) across the subjects. Supposing that the intercept in a regression model is the random parameter implies that every subject has a different intercept, and that these intercepts are assumed to be drawn from a (normal) distribution. Put simply, each subject has a different baseline. The individual baseline is represented by a separate regression line fitted through all study trials, thus providing each subject with an idiosyncratic set of parameters. This set includes—as a minimum—an individual random intercept which allows the individual means to vary while having a common slope for explanatory effects. Extending the random intercept with a random slope allows the slope to vary across subjects for a chosen experimental effect.

To be clear, these individual intercept and slope terms are not actually estimated. Rather, a (multivariate) normal distribution for the random intercept (and slopes) is assumed, and only the mean (vector) and the variance (-covariance matrix) of the assumed normal distribution are estimated. The estimated variance for a specific random effect can be viewed as the stochastic variability in the population around the overall (estimated) grand mean effect. The overall grand mean is usually referred to as the *fixed-effect* as it represents the average effect of the explanatory variable (e.g., age, sex, psychological traits etc.) on the response variable in the population. A fixed-effect that is significantly different from zero should be interpreted in the same way as a typical regression coefficient for the same explanatory variable in a standard regression model that is significantly different from zero: The expected change in the outcome variable is associated with a unit change in a predictor variable while holding the other covariates constant. Similarities with traditional methods aside, LMMs offer further advantages which are emphasized in the articles by Baayen et al. (2008) and Bagiella et al. (2000), and which will be discussed in the context of EEG studies in the following sections.

First, (longitudinal) EEG studies usually have a comparatively small number of subjects available for evaluation and/or comparison. Subject dropouts or poor EEG signal quality (i.e., too much noise) can lead to unequal numbers of observations or even considerable loss of data and thus reduced statistical power. LMM allows the inclusion of cases with missing observations and can cope with unbalanced designs more efficiently (Pinheiro and Bates 2000) because the models are fit by (restricted) maximum likelihood (Dempster et al. 1984).

A second advantage is related to the misconception that experimental manipulations trigger the same effects across all subjects. The consideration of random slopes for individual oscillatory differences across electrodes or clusters provides a means by which to estimate random variance in effect sizes while computing the fixed-treatment effect, for example.

Finally, because mixed models are regression models, they can incorporate both continuous (e.g., the whole EEG frequency spectrum) and categorical predictors (e.g., frequency band specific). These predictors may be fixed or vary across time (e.g., level of psychological parameters) with the design matrix for each subject being potentially different. Notably, this adds considerable flexibility to the modelling capabilities.

This flexibility and the possible richness of linear mixed-effects models require a thorough understanding of the data and the experimental design. Classification of a fixed or random-effect is not a trivial task, one which employs different possibilities and changes depending on the goals of the analysis (Gelman and Hill 2006). The classification implemented in this report is based on the previous work of Singmann and Bates (Bates et al. 2014; Singmann and Kellen 2017).^2^ The complexity and challenges involved in choosing the appropriate model structure, model interpretation and summaries, however, are beyond the scope of this report. These have previously been described in other fields and readers are referred to existing coverage for a comprehensive review (Bates 2010; Bates et al. 2014; Bolker 2015; Harrison et al. 2018; Pinheiro and Bates 2000; Singmann and Kellen 2017). This report will present an example using LMM for the statistical analysis of resting-state EEG data in a group that is known to be remarkably heterogeneous; namely, people who suffer from chronic tinnitus (ringing in the ear/s).

### A Brief Digression: Tinnitus

Chronic tinnitus is a common condition that affects an estimated 10–15% of the populations in the US and the European Union (Cederroth et al. 2013). Sufferers describe tinnitus as a phantom auditory perception that exists unrelated to any external sound source (Eggermont and Roberts 2004). A generally accepted concept of tinnitus generation suggests that it is preceded by damage to the cochlea with an associated hearing loss that leads to dysfunction along the auditory pathway and eventually affects the auditory cortex (Roberts et al. 2012). After the initial stage and as the condition becomes chronic, additional functional and neuroanatomical changes in non-auditory brain regions can be observed (Adjamian et al. 2009; Elgoyhen et al. 2015; Jastreboff 1990; Rauschecker et al. 2015; De Ridder et al. 2011; Vanneste and De Ridder 2012). These alterations are associated with neural processes such as increased synchronicity, hyperactivity and burst-firing (Shore et al. 2016), which are all typical for dysfunctional states of brain activity. To investigate neural activities, neuroimaging techniques such as EEG and magnetoencephalography (MEG) have been utilized in the search to identify a general blueprint which would yield insights into the tinnitus-specific modulation of ongoing neuronal oscillations compared to non-tinnitus controls (for reviews, please see Adjamian et al. 2009; Elgoyhen et al. 2015; and Zobay et al. 2015). Moreover, past findings have led to the formulation of several pathophysiological, partly contradictory, models for the generation (Hullfish et al. 2019) and manifestation of tinnitus (Noreña 2011; Rauschecker et al. 2010; De Ridder et al. 2011; Roberts et al. 2013; Sedley et al. 2016). Indeed, a clear distinction of similarities and dissimilarities between the tinnitus and non-tinnitus brain has yet to be identified, as findings have not been consistent across studies (Elgoyhen et al. 2015; Güntensperger et al. 2017). Potential reasons for the discrepancies in oscillatory findings are manifold, and may include differences in study design and sample selection; technique- and analysis-specific aspects (Adjamian 2014; Gross et al. 2013; Meyer et al. 2017); biological factors such as age (Schlee et al. 2012), tinnitus duration (Schlee et al. 2009), and degree of hearing loss (Adjamian et al. 2012); comorbidities such as hyperacusis, sleep disorders, headache, and concentration problems (Zirke et al. 2010); and associated psychopathological symptoms such as tinnitus-related distress and depression, which contribute their own levels of oscillatory correlates. Along these lines, Meyer et al. conducted a study that was explicitly designed to re-enact a former EEG resting-state experiment (Joos et al. 2012; Meyer et al. 2017). The research group was able to replicate the behavioral but not the neural findings of the reference study by Joos and colleagues. This result implies that, despite a similar pattern of behavioral results between studies, the variance in complex EEG signals may make the comparison between resembling studies almost impossible. Hence, given all the aforementioned factors (and others not been mentioned here) that contribute to the non-uniform appearance of the subjective perception of tinnitus, it is not surprising that inconsistencies across M/EEG studies are generally encountered. This culminates in the challenge on how to consider the associated aberrant oscillatory brain activity of a phenomenon, such as individual tinnitus perception, and the possible dependencies with behavioral measures in the statistical analysis of the data.

We accept the challenge with this report, the aim of which is to shed more light onto the neuronal oscillations of a heterogeneous group of subjects; namely, people who suffer from chronic tinnitus symptoms, based on (*i*) linear mixed-effect modeling and (*ii*) taking possible confounding variables, such as tinnitus duration and perceived tinnitus-related distress, into account. While we are well aware that LMM may not explain the individual oscillatory activity of tinnitus per se, the exploratory approach in this report accounts for the existing variability between individuals and hence allows us a more reliable interpretation of the data.

## Methods

### Participants

Participants in this study were part of an extensive clinical neurofeedback trial by Güntensperger et al. (2019) and were recruited at the Department of Otorhinolaryngology at the University Hospital Zurich. Prior to the intervention, resting-state EEG data were successfully collected for 49 participants (38 male, aged 24–75 years, mean age of 46.69 ± 12.65 years). Table 1 summarizes behavioral and biographical sample characteristics. Inclusion criteria at screening were as follows: adults (18–75 years) experiencing chronic subjective tinnitus (i.e., for > 0.5 years prior to the study), having sufficient command of the German language to read, understand and complete the questionnaires, as well as no other psychiatric or neurological disorder or acute suicidal tendency. Participants with drug or alcohol addiction, current prescriptions for tranquilizers, neuroleptics, or antiepileptics, and cochlear implants were not included in the study. Each participant gave their written informed consent prior to partaking in the experimental trials.

**Table 1:** Clinical characteristics

	Mean	SD	Median	Min	Max	Range
Age in years	46.69	12.65	45	24	75	51
Sex						
Female	11					
Male	38					
Tinnitus duration in months	97.67	91.71	48	8	360	352
THI score	34.04	17.68	30	4	84	80

*SD* standard deviation

### Procedure

The initial clinical trial consisted of 20 sessions to investigate the efficacy of an individualized neurofeedback protocol in the treatment of chronic tinnitus (Güntensperger et al. 2019). The interested reader is referred to the publication by Güntensperger and colleagues for an in-depth description of the procedure and the EEG recordings (2019).

The subject of the current report is the EEG and behavioral data of the two baseline sessions. In the first baseline session, participants underwent a screening interview for personal data, medical history and a standard audiometry. During the second baseline session, participants were asked to complete various questionnaires covering tinnitus-related symptoms, demographics and other psychological and health-related questions. The choice of questionnaires in the set followed the guidelines of the Tinnitus Research Initiative (TRI) (Landgrebe et al. 2012). In the study described here, the main behavioral outcome measures are tinnitus-related distress and other tinnitus-related characteristics (i.e., tinnitus duration in months). To assess the impact on lifestyle and the overall psychological distress from the phantom percept, we utilized the global score of the Tinnitus Handicap Inventory (THI, German version by Kleinjung et al. 2007). The THI was preferred because the item content predominantly assesses tinnitus distress rather than other tinnitus-related impacts (Frackell and Hoare 2014; Kennedy et al. 2004). Other tinnitus properties were collected using the adjusted version of the Tinnitus Sample Case History Questionnaire (TSCHQ), which was developed by Langguth et al. (2007) as part of a consensus for tinnitus patient assessment. In addition to the behavioral measures, we conducted resting-state EEG recordings during the second baseline session.

### EEG Recordings

Eight minutes of resting-state EEG was obtained in a soundproof and electrically shielded room using 64 active channel actiCap electrode caps coupled to a BrainAmp DC amplifier system (Brain Products GmbH, Gilching, Germany). Following the established 10/5 position system (Oostenveld & Praamstra 2001), the Ag/AgCl electrodes were set in the corresponding array. The central frontal electrode FCz was used as online reference and AFz as ground electrode. All impedances were kept below 10 kΩ. The resting-state EEG data was acquired with a sampling rate of 1000 Hz and a direct current (DC) mode with a high-cutoff filter of 1000 Hz with a slope of 12 dB/octave. Electrolyte gel was used to attain conductivity between the skin and the electrodes.

### EEG Preprocessing

For EEG data preprocessing, BrainVision Analyzer 2 (Brain Products GmbH, Gilching, Germany) was used. Continuous raw data was bandpass filtered with Butterworth zero-phase filters between 0.1 Hz and 80 Hz, with a slope of 24 dB/octave at the low cutoff and a slope of 48 dB/octave at high cutoff; as well as a 50 Hz Notch filter (bandwidth of 1 Hz, slope of 24 dB/octave) to eliminate the electrical interference. Bad channels were excluded according to standardized criteria (i.e. noise, drift or low activity). Artefact correction was performed in two steps: first, removal of eye blinks and muscular/ pulse artefact using an independent component analysis (ICA) by applying the restricted Infomax (Gradient) algorithm with classic sphering in 512 iterations and the subsequent inverse ICA procedure implemented in BrainVision Analyzer 2; second, removal of excessive artefacts following visual inspection. As a next step, spline-type topographical interpolations was used to re-implement the previously excluded channels. The recorded data were re-referenced to an averaged reference while re-including the implicit reference channel of the actual recording (i.e., FCz). In a last step, segments were created and subsequently transposed into MATLAB Statistics Toolbox (Version 2017a, The MathWorks Inc., Natick, MA, USA).

**Fig. 1:**
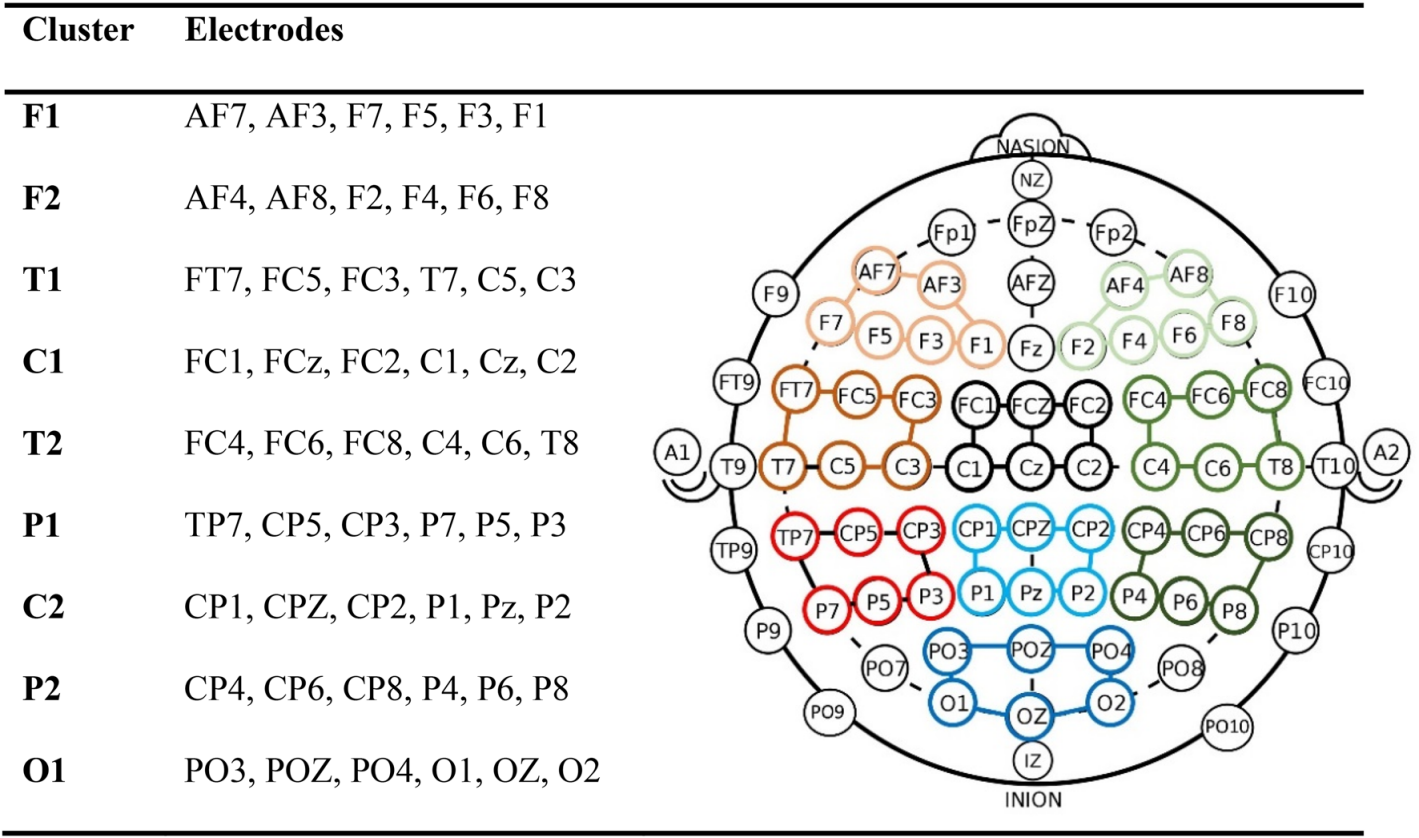
Schematic drawing of electrode montage. Each cluster consisted of six adjacent electrodes. Clusters were numbered from frontal (Cluster F1 orange; F2 light green), temporal (T1 brown; T2 forest green), central (C1 black; C2 light blue), parietal (P1 red; P2 dark green), and occipital (O1 dark blue) according to their location along the anterior–posterior dimension

EEGLAB Toolbox (Version 14.1.1b, (Delorme anbd Makeig 2004)) was used to apply a hamming window with 2 s window length and 1 s overlap. A Fast Fourier Transform (FFT) was computed for each of those 2 s-sequments, followed by a logarithmization (10 ×  log10(*x*)). As a next step, the grand average of the segments was calculated, resulting in power values in decibel (dB) for each electrode and participants. EEG power values had a frequency resolution of 0.5 Hz and were averaged for each of the 65 electrodes according to standard bands: delta (0.5–4 Hz), theta (4.5–8 Hz), lower alpha (8.5–10 Hz), upper alpha (10.5–12 Hz), beta1 (12.5–15 Hz), beta2 (15.5–23 Hz), beta3 (23.5–35 Hz), and gamma (35.5–45 Hz). For the statistical analysis, power changes were averaged across grouped electrodes. Hence, we performed our analysis with nine clusters consisting of six electrodes each (see Fig. 1). These nine clusters were selected a priori based on previously published studies of tinnitus-related distress (Vanneste et al. 2010; Weisz et al. 2005) and our own work (Meyer et al. 2014, 2017) (Fig. 1).

### Statistics

After EEG analysis, a total of 50,960 data sample points were available for statistical analysis (49 participants × 54 electrodes × 8 frequency bands). Data were analyzed using multi-level linear mixed-effects analysis with EEG power as the dependent variable. We specified a linear mixed model (LMM) with independent variables of distress (total THI score; continuous variable), frequency band (FrB; categorical variable; factor of 8 levels: δ, θ, lower-α, upper-α, β1, β2, β3, γ), and EEG cluster (categorical variable; factor of 9 levels; see Fig. 1) with interaction terms, as well as tinnitus duration (in months; continuous variable). For example, the interaction between FrB and THI is defined as the effects of FrB on EEG power, and depends on the value of THI (and vice versa). To account for repeated measures within-subject and between-subject variability, we used PatID as a clustering variable so that the effects of the frequency bands and the EEG cluster could vary across subjects. Using the formula notation in R, the full model was defined as: $$ Power \sim THI*FrB*Cluster + Duration + \left( {FrB + Cluster|PatID} \right),data, REML = FALSE, method = \hbox{''}S\hbox{''} $$

Continuous predictors (THI and duration) were mean-centered for the analysis, based on recommendations by Hox (2002). Model selection for the random-effects structure was based on the lowest Akaike information criterion (AIC) and Bayesian information criterion (BIC) value in combination with a significant model improvement using the restricted likelihood ratio test (Field et al. 2013). Non-independence of the dependent variable was determined with the intraclass coefficient (ICC1) and ICC(2) for the grouping factor of Subjects. Visual inspection of residual plots did not reveal any deviation from homoscedasticity or normality. We estimated the overall explained variance of the random-intercept and slope model with the pseudo *R*^2^ for the mixed-effects model in this study (Nakagawa and Schielzeth 2013).

*P* values were obtained using the Satterthwaite approximation (Luke 2017; Satterthwaite 1941). This method was chosen for two reasons. First the data set is relatively large. Second, the Satterthwaite approximation controls the Type I error rate just as well as other common methods like the Kenward Roger approximation (i.e., the default method in *afex*), but for complicated random-effects structures like the one in this study, it requires less RAM. Post-hoc analyses were performed to get contrasts, and the tests were adjusted using the multivariate *t* distribution (*mvt*) in the *emmeans* package. *P* values less than 0.05 were considered significant. All statistical analysis was performed in R (R Core Team 2019) using the R packages *afex* (Singmann et al. 2019), *emmeans* (Russell Lenth, 2019), *lattice* (Deepayan Sarkar, 2008), *multcomp* (Hothorn et al. 2008), and *multilevel* (Paul Bliese, 2016).

## Results

In this section, the results of the LMM analysis are presented. First, the variance and the variability of the found effects across subjects are discussed. We then focus on the fixed-effects of the model and finally, present the two- and three-way interactions.

### Variance and Variability

**Fig. 2:**
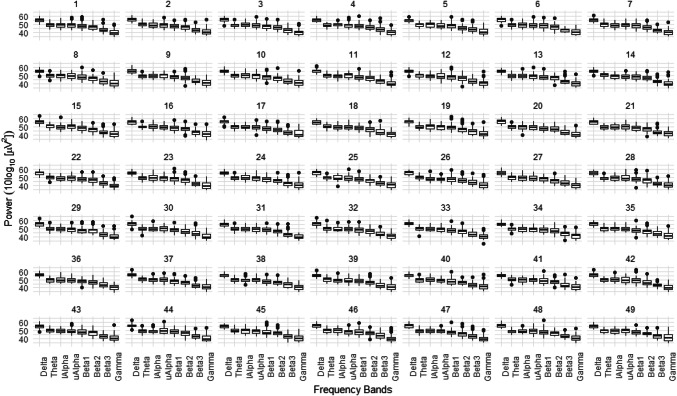
Power differences for each frequency band in all subjects. The box and whisker plots of the power differences are grouped per frequency band (delta, theta, lower-alpha, upper-alpha, beta1, beta2, beta3, gamma) for each of the 49 subjects

The random-effects are crucial for encoding measurement-dependencies in the design. The mixed model approach in this report includes individual subjects (PatID) as the random-effect grouping factor. 57.8% of the total variance in the power levels is attributed to the level of the individual subjects. Figure 2 provides insight into power differences for each subject.

The intraclass correlation coefficient (ICC) is a measure of proportion of variation in the dependent variable that occurs between-subject versus the total variation present (Fisher 1992). Further, the ICC provides information about the dependencies between observations; in this report, the individual subjects (Kreft and Leeuw 1998). Mathematically, the ICC represents a ratio of true variance over true variance plus error variance (Bartko 1966). The ICC1 is 0.124 which indicates that 12.4% of the variance of EEG power depends on the subject; non-independence is present (ICC(1) = 0.124*, F*(48, 21,119) = 64.74*, p* < 0.001). The ICC2 is usually interpreted as a measure of reliability. Using the present model, the ICC2 is 0.985, which indicates that the subjects can be reliably differentiated in terms of their average level of EEG power values.

In addition, we estimated the overall variance explanation of the model with the pseudo-*R*^2^ for mixed-effects models (Nakagawa and Schielzeth 2013). The marginal-*R*^2^ (*R*^2^_m_) considers only the variance of the fixed-effects, while the conditional-*R*^2^ (*R*^2^_c_) takes both the fixed and random-effects into account: pseudo-*R*^2^_m_ = 0.675 and pseudo-*R*^2^_c_ = 0.908.

### Fixed-Effects

Based on the results in Table 2, the next section details the estimates of the fixed-effects. Distress (THI score) and tinnitus duration were used to predict EEG activity across eight frequency bands in nine electrode clusters. A model with random intercept and slope served as the baseline model to test effects of between-subject variables on EEG power differences. We added between-subject (THI and tinnitus duration) and within-subject variables (frequency bands and EEG cluster) and included interactions of predictors. The linear mixed-effects model with EEG power as dependent variable revealed a main effect of frequency bands (*F*(7, 48.94) = 517.34*, p* < 0.0001) and cluster (*F*(8, 48.92) = 62.81, *p* < 0.0001). No effects were observed for distress (*p* = 0.59) or tinnitus duration (*p* = 0.21).

**Table 2:** Fixed-Effects ANOVA Results

Effect	*df*_Num_	*df*_Den_	*F*	*p*
FrB	7	48.94	517.34	< 0.0001***
THI	1	50.71	0.29	0.59
Cluster	8	48.92	62.81	< 0.0001***
Duration	1	49.43	1.65	0.21
FrB × THI	7	48.94	1.83	0.10
FrB × Cluster	56	20382.24	64.31	< 0.0001***
THI × Cluster	8	48.92	2.67	0.02 *
FrB × THI × Cluster	56	20382.24	1.88	< 0.0001***

*Note df*_Num_ indicates degrees of freedom numerator, df_Den_ indicates degrees of freedom denominator. *p* values for all fixed-effects are computed by Satterthwaite approximation for *df*

*p < 0.05, ***p < 0.001

Using interactions between frequency bands, clusters and distress, the analysis revealed a series of significant effects. In the following, the interactions are presented in order of increasing complexity.

### Two-Way Interaction

**Fig. 3:**
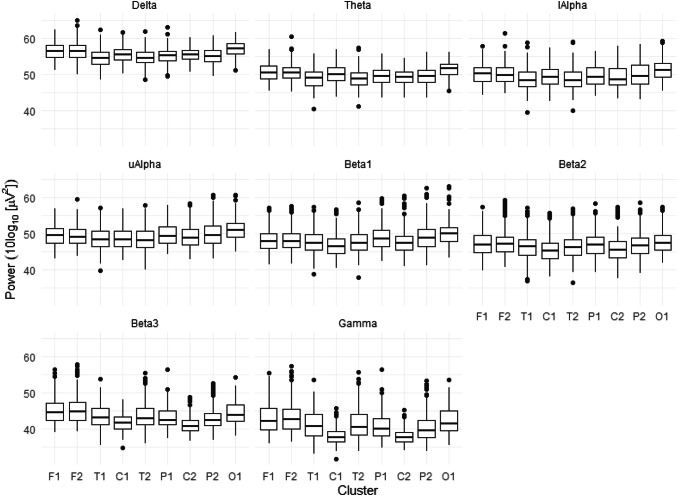
Power differences in each cluster for all frequency bands. The box and whisker plots show frequency band specific EEG power fluctuation in each EEG cluster (frontal (F1, F2), temporal (T1, T2), central (C1, C2), parietal (P1, P2), and occipital (O1)). The horizontal line in the middle of each box is the median; the lower and upper sides of the boxes are the 25th and 75th percentiles, respectively; the upper and lower whiskers were derived using the standard setting in R statistics (Chambers et al. 1983, p.62), and the dots represent outliers

The analysis of EEG power change revealed significance in the interaction between frequency band and cluster*, F*(56, 20,382.24) = 64.31,* p* < 0.0001 (for power difference in each cluster see Fig. 3). A significant cluster by distress interaction was also observed, *F*(8, 48.92) = 2,67,* p* = 0.02*,* showing that the effect on power of the level of distress (quantile steps) significantly differs over frontal regions (F1 and F2). No significant two-way interaction of Frequency Band x Distress was observed (*p* = 0.10).

### Three-Way Interaction

Adding one more interaction, the factor frequency band, to Cluster x Distress, revealed a significant three-way interaction, *F*(56,20,382.24) = 1.88*, p* < 0.0001. We observed significance of this interaction for delta in the clusters T1 (*p* = 0.032) and T2 (*p* = 0.011), whereas cluster F2 revealed a significant three-way interaction with distress and the frequency bands beta3 and gamma (*p* < 0.05). Post-hoc analysis revealed the trend of the THI score. This R function is useful when a fitted model involves a continuous predictor *x* (in this report the THI score) interacting with another predictor *a* (typically a factor, like frequency bands or cluster). Table 3 illustrates the positive THI score trends corresponding to delta in T1 and T2. The negative trend corresponding to beta3, and gamma in cluster F2 are given in Table 3. No significant differences emerged in the other frequency bands or clusters (*p* < 0.05).

**Table 3:** Post-hoc analysis of the three-way interactions: THI-score × cluster X frequency band

Cluster	Frequency band	THI trend	SE	*df*	asymp LCL	asymp UCL	*z* ratio	*p* value
Cluster = F2	Delta	0.009454	0.0142	1	− 0.01833	0.03724	0.667	0.5049
	Theta	0.000495	0.0171	1	− 0.03304	0.03403	0.029	0.9769
	lAlpha	0.002171	0.0216	1	− 0.04026	0.0446	0.1	0.9201
	uAlpha	0.014006	0.0226	1	− 0.03034	0.05835	0.619	0.5359
	Beta1	0.003682	0.0238	1	− 0.04299	0.05035	0.155	0.8771
	Beta2	− 0.013689	0.0249	1	− 0.06253	0.03515	− 0.549	0.5827
	Beta3	− **0.044725**	0.0207	1	− 0.08538	−0.00407	− 2.156	**0.0311**
	Gamma	− **0.059949**	0.0216	1	− 0.1022	− 0.0177	− 2.781	**0.0054**
Cluster = T1	Delta	**0.029221**	0.0136	1	0.00255	0.0559	2.147	**0.0318**
	Theta	0.015062	0.0173	1	− 0.01878	0.04891	0.872	0.3831
	lAlpha	0.022513	0.0219	1	− 0.02039	0.06542	1.028	0.3037
	uAlpha	0.032147	0.0231	1	− 0.01306	0.07735	1.394	0.1634
	Beta1	0.024478	0.0245	1	− 0.02348	0.07244	1	0.3172
	Beta2	0.019728	0.025	1	− 0.02922	0.06868	0.79	0.4296
	Beta3	0.007859	0.02	1	− 0.03127	0.04699	0.394	0.6939
	Gamma	− 0.001512	0.0206	1	− 0.04195	0.03893	− 0.073	0.9416
Cluster = T2	Delta	**0.027582**	0.0134	1	0.00124	0.05392	2.052	**0.0401**
	Theta	0.016042	0.0168	1	− 0.01691	0.04899	0.954	0.3399
	lAlpha	0.020857	0.0215	1	− 0.02137	0.06309	0.968	0.333
	uAlpha	0.027937	0.0227	1	− 0.01651	0.07239	1.232	0.218
	Beta1	0.0198	0.0237	1	− 0.02668	0.06628	0.835	0.4038
	Beta2	0.007942	0.0248	1	− 0.04072	0.05661	0.32	0.7491
	Beta3	− 0.018403	0.0209	1	− 0.05935	0.02254	− 0.881	0.3784
	Gamma	− 0.03455	0.022	1	− 0.07758	0.00848	− 1.574	0.1156

Bold values indicate statistical significance (*p* < 0.05)

*Note* Post-hoc analysis with the emtrends function by the package emmeans. It is useful when a fitted model involves a numerical predictor x interacting with another predictor a (typically a factor). Such models specify that x has a different trend depending on a. Thus, in cluster F2 the THI score has a small negative trend for beta3 and gamma starting from their averaged values. Delta in Cluster T1 and T2 corresponds to a small increase in the THI score. Confidence level used: 0.95

Taken together, our results indicate that change of the perceived distress measured with the Tinnitus Handicap Inventory resulted in oscillatory alteration at all nine regional sources (clusters). However, only over the right frontal (F2) and temporal-parietal (T1, T3) regions were significant effects of distress observed. In relationship with all frequency bands (delta, l-alpha, u-alpha, beta1, beta2, beta3, and gamma), the perceived distress targeted significantly delta, beta3 and gamma oscillations. Further, EEG power differs between individuals, which indicates that there are inter-individual differences in the found effects and in the pattern of EEG power oscillations. The fact that these inter-individual differences can be modeled and quantified illustrates the added value of using LMM in EEG studies.

## Discussion and Conclusion

To study spontaneous brain activity of a population or to contrast between two samples, the distinctions between individuals or the heterogeneity within the group must be considered. We investigated the resting-state neural oscillations of a group of tinnitus sufferers with the aim of statistically acknowledging the individual differences in the subjective tinnitus experience while, at the same time, taking into account the similarities in tinnitus perception. Here, the cardinal individual tinnitus symptom (i.e., the tinnitus-related distress) was quantified by the Tinnitus Handicap Inventory score. The reported findings suggest that oscillations are associated with tinnitus-related distress in the delta band over temporal-parietal regions, and beta3 and gamma in the right frontal lobe, findings that are consistent with previous observations (Adjamian et al. 2012; Meyer et al. 2017 Schlee et al. 2008;Vanneste et al. 2010; and Weisz et al. 2005). Also in line with these former studies, the individual’s subjective level of tinnitus distress is reflected by changes in the neural patterns over non-auditory regions, such as the frontal lobes. Frontoparietal networks are comprised of higher-order association cortex rather than primary sensory regions as is the case in the primary auditory cortex. These cortical associative regions, which have been related to learning, attentional and emotional processes, are also the most recent to develop in an evolutionary sense (Zilles et al. 1988), and show the highest structural and functional inter-individual variance (Miranda-Dominguez et al. 2014; Mueller et al. 2013).

Examining the idiosyncrasy of the EEG oscillations, the present results indicate that some of the recorded EEG power variance can be attributed to the individual subject. In more detail, spontaneous brain signals derived from a single subject in a single recording (and/or across a certain period of time) are more likely to be similar to each other than two recordings coming from two different individuals. Further, data obtained from individuals can be reliably differentiated, a finding which implies consistency of the individual’s average level of EEG power values. Thus, for data with repeated measurements per person, the iid assumption with respect to the residuals is likely to be violated. This makes it more important to efficiently model the different dependencies and variabilities between- and within-subjects, and indicates the use of mixed models. The reliable attribution of some of the total variability to differences between individual subjects, and furthermore, to quantify those differences, is informative in its own right (Barr et al. 2013; Matuschek et al. 2017). A possible limitation in this context might be the high demand for data to achieve robust estimates of the random-effects variance (Gelman und Hill 2006). To properly estimate the variance, LMM requires more than five levels for the random-effect term. To illustrate, the effect of subjects with a minimum of five individuals would be an adequate random-effect term, whereas the effect of sex as a two level factor of male and female could only be assigned as a fixed-effect. The underlying assumption of a mixed-model is that the levels are a random sample out of a population (as in our example, the subjects in a study are a random sample of a population). Therefore, it is typically assumed that the effects follow a normal distribution in the population, for which only the mean and variance are estimated, rather than the individual effects. For sex, there are only two possibilities in this study, so the assumption that sex is normally distributed in the population is not sensible—the effect of sex can simply be estimated. In contrast, the individual effects of all the subjects in a study would lead to a very high number of additional parameters needing to be estimated. By assuming normally distributed effects, only the mean and variance of the effect are estimated.

If one considers this limitation in combination with the advantages of this modeling approach, such as handling unequal observations, accounting for variability in effect size across individuals, as well as flexibility in designs, LMM enables a more transparent overall prediction to be obtained. Thus, a deeper understanding of both the underlying statistical and physiological patterns of EEG derived data is gained.

Through our presentation and discussion of a fairly simple and tinnitus-focused example of mixed-effects modeling, this report provides a generally informative introduction to and application of the powerful tool of LMMs. The collections of references provide an overview of the primary literature and showcase the potential for this statistical tool to be implemented in more complex M/EEG study designs from a range of domains. Specifically, any scientific studies in which the hierarchical non-independency of input space is attributed to the individuality of a subject. More generally speaking, in cases in which heterogeneous groups of subjects are mixed, the statistical modeling approach of LMM could be used to account for subtle changes in oscillatory activity, to validate existing theoretical models, to elucidate the source of at least parts of the reproducibility issues, and to focus on the relevant theoretical issues in M/EEG research.

## Footnotes

1 The independence assumption refers to the residuals: After taking the structure of the model (i.e., parameters) into account, the probability of a data point having a specific value is independent of all other data points. For an identical distribution, all observations are sampled from the same distribution.

2 Green and Tukey provide an alternative definition: “When a sample exhausts the population, the corresponding variable is fixed; when the sample is a small (i.e., negligible) part of the population the corresponding variable is random” (1960)
